# Novel compound heterozygous variants of *SLC12A3* gene in a Chinese patient with Gitelman syndrome: a case report

**DOI:** 10.3389/fgene.2023.1067242

**Published:** 2023-06-12

**Authors:** Wenqing Chen, Qin Zhou, Hongjun Chen, Heng Li, Jianghua Chen

**Affiliations:** ^1^ Kidney Disease Center, The First Affiliated Hospital, College of Medicine, Zhejiang University, Hangzhou, China; ^2^ Key Laboratory of Kidney Disease Prevention and Control Technology, Hangzhou, Zhejiang, China; ^3^ Institute of Nephropathy, Zhejiang University, Hangzhou, China; ^4^ Zhejiang Clinical Research Center of Kidney and Urinary System Disease, Hangzhou, China

**Keywords:** Gitelman syndrome, clinical characteristics, *SLC12A3* gene, whole-exome sequencing, gene mutation

## Abstract

**Background:** The Gitelman syndrome (GS) is an autosomal recessive disorder of renal tubular salt handling. Gitelman syndrome is characterized by hypokalemia, metabolic alkalosis, hypomagnesemia, hypocalciuria, and renin-angiotensin-aldosterone system (RAAS) activation, and is caused by variants in the *SLC12A3* gene. Gitelman syndrome has a heterogeneous phenotype, which may or may not include a range of clinical signs, posing certain difficulties for clinical diagnosis.

**Case presentation:** A 49-year-old man was admitted to our hospital due to muscular weakness. The patient’s history revealed previous recurrent muscular weakness events associated with hypokalemia, featured by a minimum serum potassium value of 2.3 mmol/L. The reported male patient had persistent hypokalemia, hypocalciuria and normal blood pressure, without presenting obvious metabolic alkalosis, growth retardation, hypomagnesemia, hypochloremia or RAAS activation. We performed whole-exome sequencing and identified a novel compound heterozygous variant in the *SLC12A3* gene, c.965-1_976delGCGGACATTTTTGinsACCGAAAATTTT in exon8 and c.1112T>C in exon9 in the proband.

**Conclusion:** This is a study to report a heterogeneous phenotype Gitelman syndrome with a novel pathogenic compound heterozygous variant in the *SLC12A3* gene*.* This genetic study expands the variants spectrum, and improve the diagnostic accuracy of Gitelman syndrome. Meanwhile, further functional studies are required to investigate the pathophysiological mechanisms of Gitelman syndrome.

## Introduction

Gitelman syndrome (GS) (OMIM 263800) is an inherited autosomal recessive salt-losing tubulopathy caused by biallelic inactivating variants in the *SLC12A3* gene encoding a thiazide-sensitive sodium-chloride cotransporter (NCC), which is exclusively expressed in the apical membrane of cells lining the distal convoluted tubule (DCT) (Reyes and Medina, 2022). With a prevalence of approximately 1–10 per 40,000 cases, which is potentially higher in Asia. GS is an autosomal recessive disorder, but homozygous variants are found in only 18% of patients (Hsu et al., 2009; Blanchard et al., 2017; Wang et al., 2020). More than 45% of GS cases featured compound heterozygous variants, 30% had single heterozygous variants, and 7% exhibited three or more variants (Lee et al., 2016; Blanchard et al., 2017). The gender effect on phenotype in GS has been reported, with male patients carrying compound heterozygous variants leading to splicing defects and intrinsic functional alterations in NCC exhibiting severe phenotypes (Riveira-Munoz et al., 2007). Several variants in *SLC12A3* and *CLCNKB* were identified in patients, and associated with clinical phenotypes in patients with GS and Gitelman-like syndrome (Lee et al., 2016; Kong et al., 2019). Here, we report a male patient with GS, characterized by a novel compound heterozygous variant of the *SLC12A3* gene.

## Materials and methods

### Whole exome sequencing

Genomic DNA (gDNA) of patient was extracted from the peripheral blood using MagPure Buffy Coat DNA Midi KF Kit (Magen, China). Then, gDNA was broken into 100–500 bp fragments using enzyme kit (BGI, China), 280–320 bp fragments were collected by magnetic beads. Agilent 2100 bioanalyzer and BMG were used to estimate the enrichment degree, and qualified products were collected to make DNA nanoballs and quantified according to different library quantities. DNA nanoballs were sequenced with PE100 + 100 on MGISEQ-2000. The average sequencing depth of the target region is ≥ 200×, more than 96% of the locus have a coverage depth of >20×.

## Case presentation

### Clinical history and laboratory data

A 49-year-old male was presented to our hospital with the major complaints of fatigue, polydipsia, polyuria, and repeated muscle weakness, but without a history of salt craving, constipation, physical, and intellectual disability over a 30-year period. Due to the diagnosis of renal tubular acidosis 25 years earlier, the patient had token potassium sodium hydrogen citrate granules orally for a long time to maintain the blood potassium at 2.0–2.5 mmol/L. Two months earlier, the patient was admitted to the endocrinology department due to fatigue and developed hypokalemia. The patient was supplied with potassium chloride sustained release tablets. During the follow-up, serum potassium levels were maintained between 2.3 and 2.55 mmol/L. He was hospitalized in our department due to recurrent fatigue.

The patient had a height of 169 cm, weight of 69 kg, and a heart rate of 78 beats per minute with no growth retardation. His respiratory rate was 18 breaths per minute, temperature was 37.0°C, and 24-h urine volume was 1.9–2.4 L. We monitored the blood pressure of this patient twice a day, with the systolic blood pressure in the range of 95–123 mmHg, and the diastolic blood pressure in the range of 60–76 mmHg. The laboratory investigation revealed hypokalemia, hypocalciuria and normal blood pressure, without obvious metabolic alkalosis, hypomagnesemia, hypochloremia or RAAS activation (Table 1). The fractional excretion rate of potassium (FEK%) was significantly increased to 30.25% (normal range, 8%–12%) and spot potassium-creatinine ratio elevated to 7.33, suggesting that hypokalemia resulted from renal potassium loss (Table 1). Urinalysis showed microalbuminuria, normal urinary immunoglobulin IgG, elevated β2-microglobulinuria, and elevated urinary retinol binding protein, which indicated proximal tubular injury. Other possible causes of hypokalemia, such as thyrotoxic periodic paralysis, renal tubular acidosis, and hypercortisolism, were excluded. The enhanced computed tomography of adrenal gland showed possible right adrenal myelolipoma and left adrenal outer limb nodular protrusion, and hyperplasia was also considered (Supplementary Figure S1). Nevertheless, the renin-angiotensin-aldosterone system was normal, hence primary aldosteronism was excluded.

**TABLE 1:** Biochemical characteristics of the proband.

Variable	Test value	Reference range
Blood tests		
Potassium (mmol/L)	2.09	3.50–5.30
Sodium (mmol/L)	148	137–147
Chloride (mmol/L)	105	99–110
Calcium (mmol/L)	2.00	2.11–2.52
Phosphate (mmol/L)	0.46	0.85–1.51
Magnesium (mmol/L)	0.97	0.75–1.02
Urea nitrogen (mmol/L)	3.38	3.1–8.00
Creatinine (μmol/L)	102	57–97
Fasting blood glucose (mmol/L)	4.45	3.90–6.10
Uric acid (μmol/L)	302	208–428
PTH(pg/mL)	9.6	15–65
ACTH (8AM) (pg/mL)	30	0.00–46.00
Renin–angiotensin–aldosterone system		
Renin (upright) (μIU/mL)	42.1	4.4–46.1
Aldosterone (upright (pg/L)	147	30–353.0
ARR	3.49	
Renin (supine) (μIU/mL)	17.3	2.8–39.9
Aldosterone (supine) (pg/L)	115	30–236.0
Arterial blood gas analysis		
PH	7.408	7.35–7.45
pCO_2_	39.7	35–45
Bicarbonate (mmol/L)	26.9	22–27
Urine tests		
PH	8.0	4.5–8.0
Urine protein (g/L)	±(0.15)	-
MAU (g/mol.Cr)	0.890	0.000–3.000
Urinal β2 microglobulin (g/mol.Cr)	2.478	0.000–0.045
URBP(g/mol.Cr)	1.305	0.000–0.105
UACR (g/g)	0.23	0–0.20
Urinary aldosterone (μg/24 h)	5.442	1.0–8.0
urinary free cortisol (μg/24 h)	260.4	20.9–292.3
Calcium (mmol/24 h)	1.9	2.5–7.5
Potassium (mmol/24 h)	80	25–100
Magnesium (mmol/24 h)	5.52	2.50–8.50
Sodium (mmol/24 h)	365	130–260
Chloride (mmol/24 h)	327	170–250
Phosphate (μmol/24 h)	11.8	12.9–42.0
spot potassium-creatinine ratio	7.33	>2.0 mmol/mmol
spot calcium-creatinine ratio	0.16	<0.2 mmol/mmol
FEMg%	4.64%	>4%
FECL%	6.79%	>0.5%

**FIGURE 1:**
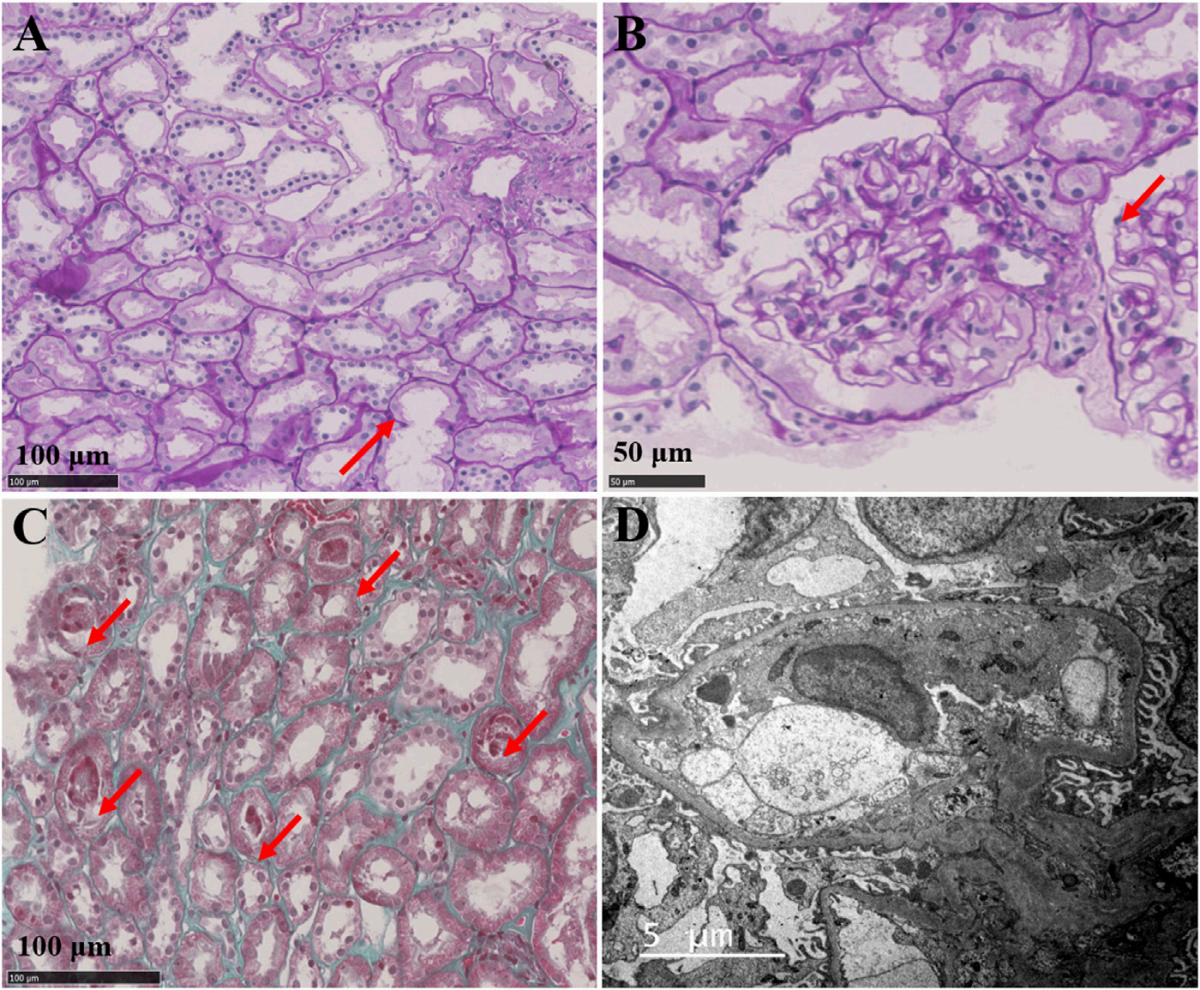
Renal pathology **(A)** Light microscope observations of PAS staining showing the focal granular and vacuolar degeneration of tubular epithelial cells; the brush border of some renal tubules fell off and the lumen expanded (arrow), and no special lesions were found in renal interstitium **(B)** Light microscope observations of PAS staining showing that the juxtaglomerular apparatus proliferation was not obvious (arrow) **(C)** Light microscope observations of MASSON staining showing exfoliated tubular epithelial cells and protein tubular formation in some kidney tubules (arrow) **(D)** Electron microscopy showing no obvious abnormalities, electron dense deposits or significant GBM thickening (280–450 nm).

The electrocardiogram (ECG) showed normal sinus rhythm, abnormality of T-waves but no prolongation of the QT interval. The urinary ultrasound showed kidneys with normal size and without obvious abnormality in the ureters (Supplementary Figure S2). The pure tone audiometry of the patient was normal. The ophthalmology examination indicated that sclerochoroidal calcifications could be excluded. A renal biopsy was performed, and the renal pathology revealed a mild injury of the renal tubular epithelium (Figure 1). The light microscope showed focal granular degeneration, cell swelling, brush edge falling off, and without tubular atrophy and tubulitis. Vacuolar degeneration of the renal tubular epithelial cells was observed, and no special lesions were found in the renal interstitium.

### Diagnosis and treatment

The patient was diagnosed with GS based upon clinical features and biochemical parameters according to the criteria (Blanchard et al., 2017), whereas the confirmation of clinically suspected GS rested on genetic testing. The blood potassium was maintained at 3.3–3.5 mmol/L by clinical follow-up, with the addition of 3 g potassium chloride tablets in three divided doses and 40 mg spironolactone in two divided doses. Subsequently, the patient’s symptoms dramatically improved.

### Whole exome sequence results

**FIGURE 2:**
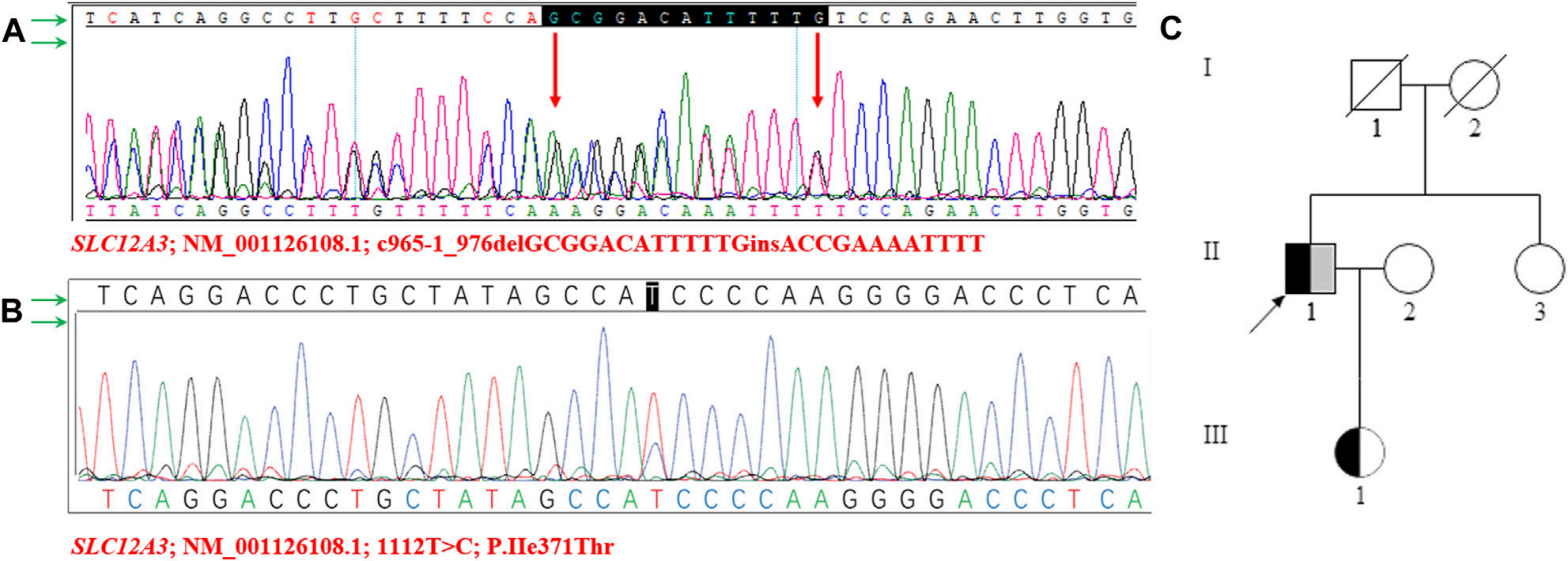
Sanger sequencing diagram of *SLC12A3* gene and pedigree of the family structure **(A–B)** The proband’s NGS revealed two heterozygous variants in the *SLC12A3* gene (c.1112T>C and c.965-1_976delGCGGACATTTTTGinsACCGAAAATTTT), which were confirmed by sanger sequencing **(C)** The pedigree of the family structure. The marked symbols show patients who carried compound heterozygous variants of *SLC12A3*. The variants of c.1112T>C were presented in black, and c.965-1_976delGCGGACATTTTTGinsACCGAAAATTTT was shown in grey. Circles present females, and squares present males. The arrow shows proband. The deceased parents of proband and II-1 showed normal phenotypes, without any features of Gitelman syndrome.

For a precise diagnosis, we performed whole-exome sequencing and identified a novel compound heterozygous variant in the *SLC12A3* gene. One of the variants, *SLC12A3* (NM_001126108.1, c.965-1_976delGCGGACATTTTTGinsACCGAAAATTTT) is caused by the deletion of 13 nucleotides GCGGACATTTTTG, and the insertion of 12 nucleotides ACCGAAAATTTT at nucleotide positions 965-976 of the coding sequence. Another variant *SLC12A3* (NM_001126108.1, c.1112T>C, p.Ile371Thr) is caused by the substitution of nucleotide T with C. Sanger sequence were used to verify the mutations (Figures 2A, B).

Meanwhile, the sister of the proband had normal manifestations, no variants were found. Notably, a heterozygous variant (NM_001126108.1, c.965-1_976delGCGGACATTTTTGinsACCGAAAATTTT) was identified in the *SLC12A3* gene in the proband’s daughter. The daughter is 24-year-old, appeared healthy, was found to have normal plasma potassium (4.2 mmol/L) and normal serum creatinine (67 μmol/L) levels. The serum potassium, sodium, chloride, calcium, and magnesium levels of the family members were unremarkable. The pedigree structure of the family was drawn according to the clinical manifestations and the sequencing results (Figure 2C).

### Functional changes predictive of c.1112T>C variants

**FIGURE 3:**
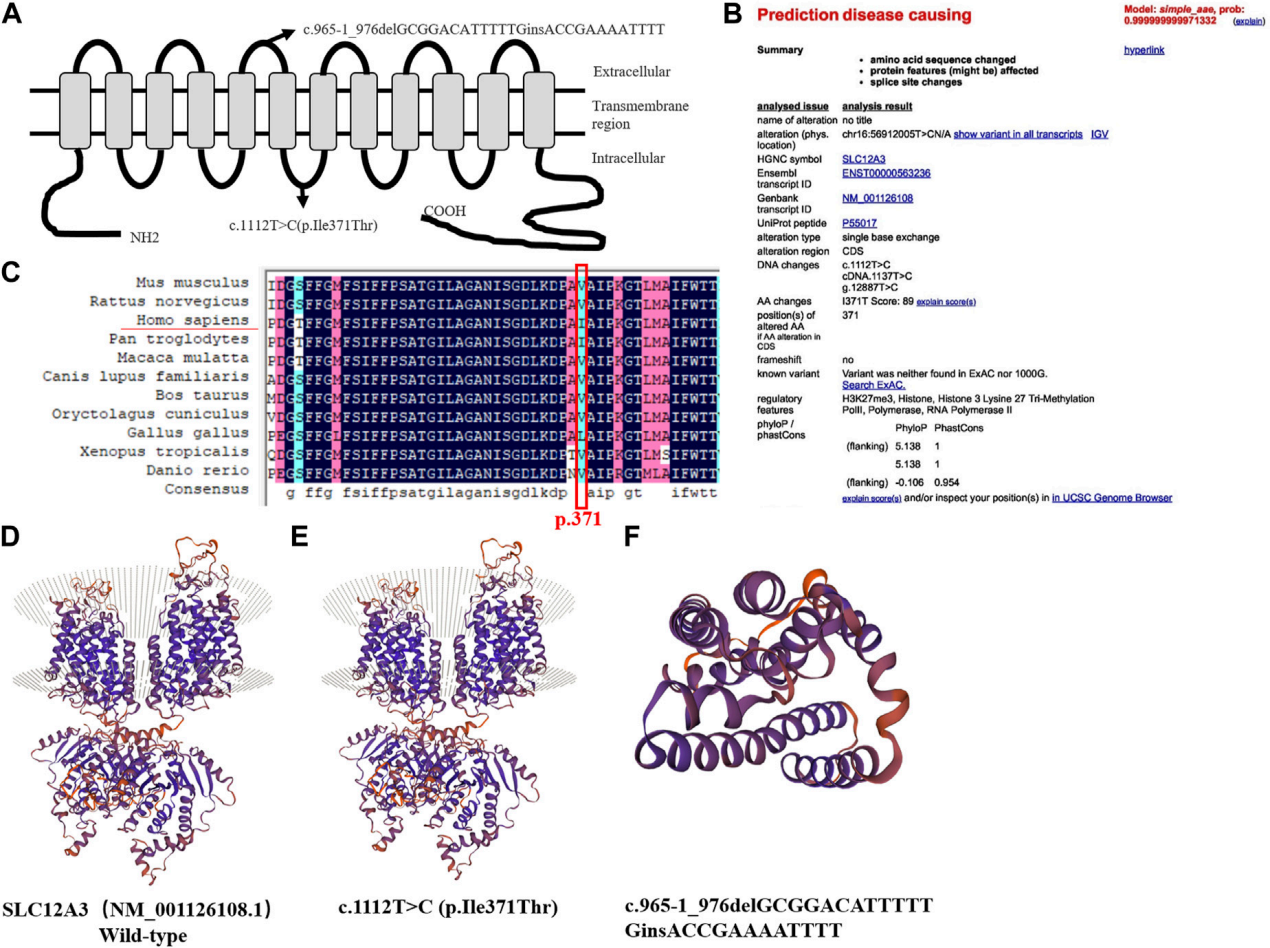
Predicted topological localization of NCC variants and effect of *SLC12A3* variants on the modeled structure of NCC protein, evaluation of the pathogenic potential of c.1112T>C variant in *SLC12A3* **(A)** Schematic diagram of NCC protein with the intercellular N- and C-terminal domains and transmembrane segments. The sites of detected variants are denoted by arrows in our study **(B)** The web-based software Variant Taster showed that this variant was disease causing, and the variant at this position was reported **(C)** Analysis of amino acid conservation based on the NCBI database. Evolutionary conservation of Ile371Thr using the ClustalX program. The black frame marks the Ile371Thr site **(D–E)** Compared to the wild-type, c.1112T>C (p.Ile371Thr) is only a point variant, with no significant change in the protein sequence **(F)** c.965-1_976delGCGGACATTTTT causes nonsense mediated mRNA degradation (NMD), because the protein sequence becomes shorter due to the early appearance of the stop codon, hence the structure is smaller.

Variants were located in the intracellular and extracellular carboxyterminal domain of the NCC protein (Figure 3A). The variant (NM_001126108.1, c.965-1_976delGCGGACATTTTTGinsACCGAAAATTTT) in the *SLC12A3* gene is a known GS associated variant (Shao et al., 2008; Wang et al., 2021). However, the variant *SLC12A3* (NM_001126108.1, c.1112T>C, p.Ile371Thr) is not included in the databases (HGMD Professional and ClinVar). According to the American College of Medical Genetics and Genomics (ACMG) guidelines, the variation was judged to be of undetermined significance. The web-based software Variant Taster predicted that this variant was disease causing and capable of triggering amino acid sequence changes, frameshift, and splice site changes (Figure 3B). Two pathogenic in-trans variants in a single gene can produce the phenotype of this disease. Since the daughter of the proband carries only one of the two *SLC12A3* variants, we can assume that these two variants are in-trans in the proband. In addition, we used CADD to predict the variant *SLC12A3* (NM_001126108.1, c.1112T>C, p.Ile371Thr). The CADD-phred score is 26.8. We used mutation taster online website to predict and find that the variant is disease causing, and the prob is 0.999999999971332 (Figure 3B). Thus, the two variants may support the GS phenotype.

### Three-dimensional structure prediction of SLC12A3 protein

Sequence alignment of SLC12A3 protein revealed that the isoleucine of p.371 was conserved among different species (Figure 3C). We used bioinformatics techniques to perform protein function prediction and secondary structure simulation (Supplementary Table S1). We used the SWISS-MODEL workspace (http://swissmodel.expasy.org) to characterize the effects of the novel variants, *SLC12A3* (NM_001126108.1, c.965-1_976delGCGGACATTTTTGinsACCGAAAATTTT) and (NM_001126108.1, c.1112T>C, p.Ile371Thr) on the protein structure of NCC, which demonstrated that the alterations caused by the variants modified the protein structure, and might even affected the function in terms of NCC physiology. The variant *SLC12A3* (NM_001126108.1, c.1112T>C, p.Ile371Thr) is only a point variant, with no significant change in the protein structure compared to wild type (Figures 3D, E). *SLC12A3* (NM_001126108.1, c.965-1_976delGCGGACATTTTTGinsACCGAAAATTTT) cause protein structure changes into a smaller structure, may affect SLC12A3 protein function (Figure 3F).

## Discussion

GS is characterized by hypokalemic metabolic alkalosis, hyperreninemia and hyperaldosteronemia. An adult patient subject to our case study was found with hypokalemia due to fatigue, and had been misdiagnosed as renal tubular acidosis for a long period. The long-term use of potassium sodium hydrogen citrate granules was insufficient for treatment. Based on the patient’s medical history and laboratory tests, the condition was conjectured to be GS with a heterogeneous phenotype.

Patients with GS with homozygous or compound heterozygous variants in *CLCNKB* and *SLC12A3* were reported in many studies, and variants in *CLCNKB* in particular seem to be responsible for a mixed Bartter-Gitelman phenotype or at least are involved in a switch in the clinical phenotype (Zelikovic et al., 2003). Thus far, there has been no report of GS associated with compound heterozygous variants in *SLC12A3*. With a compound heterozygous variant in *SLC12A3*, our patient’s clinical manifestations were consistent with a heterogeneous phenotype GS in this study.

Features in an individual GS patient may vary. Patients are often asymptomatic or present with symptoms such as muscle weakness, fatigue, salt craving, thirst, nocturia, constipation, cramps, carpopedal spasms, or tetanic episodes triggered by hypomagnesemia (Blanchard et al., 2017). The presence of both hypocalciuria and hypomagnesemia is highly predictive of the clinical diagnosis of GS, although hypocalciuria is extremely variable and hypomagnesemia may be absent (Tseng et al., 2012). Some previous studies reported normomagnesemia in GS patients (Liu et al., 2016; Ma et al., 2016; Wang et al., 2017), which indicates that patients with normal magnesium exhibit milder clinical manifestations than hypomagnesemic patients (Jiang et al., 2014). Our patient’s blood magnesium levels were normal and without RAAS activation. Low to normal plasma aldosterone concentration in male patients may reflect the suppressive effect of aldosterone by more severe hypokalemia (Tseng et al., 2012). It has been confirmed in the phenotype analysis that male patients had more severe hypokalemia and associated neuromuscular symptoms than females (Riveira-Munoz et al., 2007; Tseng et al., 2012).

Research suggests that GS may be associated with proteinuria, and chronic kidney disease might develop in GS patients due to either chronic hypokalemia, which is associated with tubulointerstitial nephritis, tubule vacuolization and cystic changes, or volume depletion and increased reninangiotensin-aldosterone, which may contribute to renal damage and fibrosis (Blanchard et al., 2017). Our patient presented a low level of proteinuria, which included elevated β2-microglobulinuria and urinary retinol binding protein, indicating proximal tubular injury. Moreover, the renal biopsy showed tubulointerstitial injury, without obvious parabulbar organ hyperplasia.

In conclusion, this paper presented a case of novel compound heterozygous variants in the *SLC12A3* gene (c.1112T>C and c.965-1_976delGCGGACATTTTTGinsACCGAAAATTTT) in a 49-year-old Chinese male with persistent hypokalemia, hypocalciuria, metabolic alkalosis and normal blood pressure, but without obvious metabolic alkalosis, hypomagnesemia, hypochloremia, or RAAS activation. The GS phenotype was complicated; accordingly, symptoms of GS are usually nonspecific and variable. This case expands the variants spectrum, and improve the diagnostic accuracy of GS. Thus far, the molecular basis of the phenotype variability in GS remains unknown, and it is not clear whether it has a critical role in managing patients and predicting their prognosis, hence further studies are needed to clarify the underlying mechanism.

## Data Availability

The original contributions presented in the study are included in the article’s Supplementary Material, further inquiries can be directed to the corresponding authors.
