# Supplementary material for: Novel compound heterozygous variants of SLC12A3 gene in a Chinese patient with Gitelman syndrome: a case report
Source: Front Genet. 2023 Jun 12;14:1067242. doi: 10.3389/fgene.2023.1067242 (PMC10291089; doi:10.3389/fgene.2023.1067242)
Supplement: Supplementary file 1 [file Table1.docx]

**Supplementary Table S1 Bioinformatics analysis of the novel compound heterozygous mutations**

| Mutated genes | Chromosome | Nucleotide | Amino Acid | ACMG | subregion | SpliceAI | PhyloP Vertebrates | PhyloP  Placetal Mammals |
| --- | --- | --- | --- | --- | --- | --- | --- | --- |
| SLC12A 3 | chr16:56912005 | c.1112T>C | p.Ile371Thr | undetermined significance | EX9/  CDS9 | [polymorphism](https://fanyi.so.com/#polymorphism) | conservation | conservation |
| SLC12A 3 | chr16:56906567-56906579 | c.965-1_976delGCGGACATTTTTGinsACCGAAAATTTT | - | Disease causing | IVS7-EX8/  AC7-C8 | - | - | - |
| BSND | chr1:55472714 | c.318delC | p.Tyr107Metfs*13 | Disease causing | EX3/  CDS3 | [polymorphism](https://fanyi.so.com/#polymorphism) | conservation | conservation |
