# Supplementary material for: Novel compound heterozygous variants of SLC12A3 gene in a Chinese patient with Gitelman syndrome: a case report
Source: Front Genet. 2023 Jun 12;14:1067242. doi: 10.3389/fgene.2023.1067242 (PMC10291089; doi:10.3389/fgene.2023.1067242)
Supplement: Supplementary file 2 [file Image2.pdf]

A

B

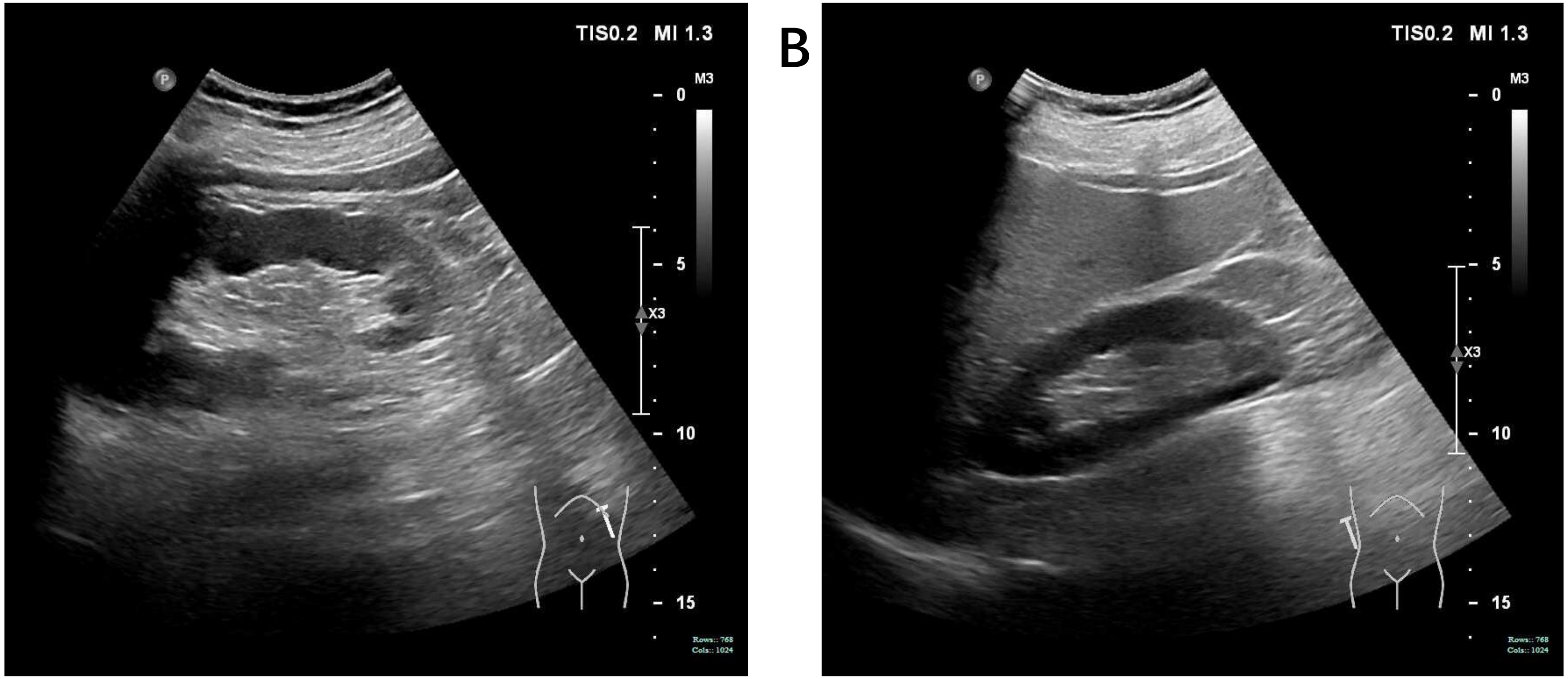

A. the left kidney

B. the right kidney

Supplementary Figure S2

Urinary ultrasound showed that the size of the left kidney was about 10.3 * 4.6cm, the size of the right kidney was about 10.4 * 4.1cm, without obvious abnormality in the ureters of both kidneys.
