# Supplementary material for: Novel compound heterozygous variants of SLC12A3 gene in a Chinese patient with Gitelman syndrome: a case report
Source: Front Genet. 2023 Jun 12;14:1067242. doi: 10.3389/fgene.2023.1067242 (PMC10291089; doi:10.3389/fgene.2023.1067242)
Supplement: Supplementary file 3 [file Image1.pdf]

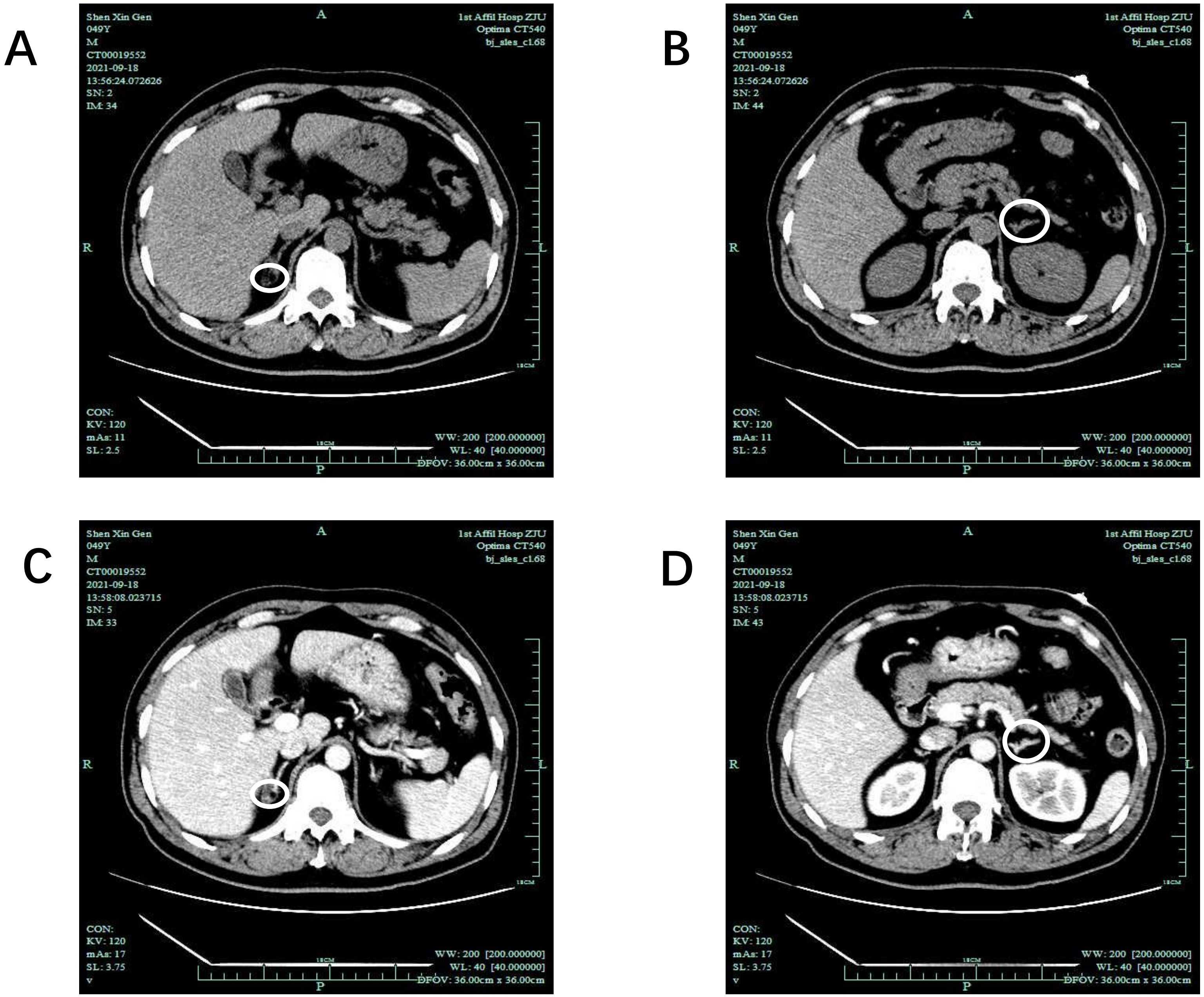
A
Shen Xin Gen
049Y
M
CT00019552
2021-09-18
13:56:24.072626
SN: 2
IM: 34
A
1st Affil Hosp ZJU
Optima CT540
bj_sles_c1.68
R
L
CON:
KV: 120
mAs: 11
SL: 2.5
WW: 200 [200.000000]
WL: 40 [40.000000]
DFOV: 36.00cm x 36.00cm
P
B
Shen Xin Gen
049Y
M
CT00019552
2021-09-18
13:56:24.072626
SN: 2
IM: 44
A
1st Affil Hosp ZJU
Optima CT540
bj_sles_c1.68
R
L
CON:
KV: 120
mAs: 11
SL: 2.5
WW: 200 [200.000000]
WL: 40 [40.000000]
DFOV: 36.00cm x 36.00cm
P
C
Shen Xin Gen
049Y
M
CT00019552
2021-09-18
13:58:08.023715
SN: 5
IM: 33
A
1st Affil Hosp ZJU
Optima CT540
bj_sles_c1.68
R
L
CON:
KV: 120
mAs: 17
SL: 3.75
v
WW: 200 [200.000000]
WL: 40 [40.000000]
DFOV: 36.00cm x 36.00cm
P
D
Shen Xin Gen
049Y
M
CT00019552
2021-09-18
13:58:08.023715
SN: 5
IM: 43
A
1st Affil Hosp ZJU
Optima CT540
bj_sles_c1.68
R
L
CON:
KV: 120
mAs: 17
SL: 3.75
v
WW: 200 [200.000000]
WL: 40 [40.000000]
DFOV: 36.00cm x 36.00cm
P

Supplementary Figure S1

Enhanced computed tomography of adrenal gland. Possible right adrenal myelolipoma on the routine scan (A) and enhanced scan (C). Left adrenal nodular protrusion was found by the routine scan (B) and enhanced computed tomography (D).
